# Diagnostic performance of desmopressin stimulation test in pediatric cushing’s disease

**DOI:** 10.1007/s11102-025-01610-4

**Published:** 2025-12-20

**Authors:** Yetunde B. Omotosho, Richard Chang, Michael Kassin, Erna Groat, Alan Quillian, Ruth Parker, Christina Tatsi

**Affiliations:** 1 Unit on Hypothalamic and Pituitary Disorders, Eunice Kennedy Shriver National Institute of Child Health and Human Development, National Institutes of Health, 10 Center Drive, Building 10, Rom 1-3330, MSC1103, Bethesda, MD 20892 USA; 2 Endocrine and Venous Services Section, Interventional Radiology Section, Clinical Center, National Institutes of Health, Bethesda, MD 20892 USA; 3 Department of Pharmacy, Clinical Center, National Institutes of Health, Bethesda, MD 20892 USA; 4 Department of Pediatrics, Clinical Center, National Institutes of Health, Bethesda, MD 20892 USA

**Keywords:** Desmopressin test, CRH test, DDAVP, Cushing’s disease, Dynamic tests

## Abstract

**Objective:**

To evaluate the diagnostic performance of the desmopressin (DDAVP) stimulation test in pediatric patients with Cushing’s disease (CD), and to compare its accuracy and safety profile to the ovine corticotropin-releasing hormone (oCRH) stimulation test.

**Design:**

A retrospective cohort study.

**Methods:**

Pediatric patients with CD who underwent peripheral or bilateral inferior petrosal sinus sampling (BIPSS) stimulation testing with either DDAVP or oCRH were included. Patients were matched 1:1 for age, sex, and tumor size. The performance of each test was assessed by evaluating ACTH and cortisol responses and calculating test sensitivities.

**Results:**

In peripheral stimulation testing, DDAVP demonstrated 96.9% sensitivity for cortisol and 81.3% for ACTH, while oCRH showed 93.8% and 96.9% sensitivities respectively (*p* > 0.05). Percentage change of ACTH was higher in the CRH group compared to DDAVP. In BIPSS, the DDAVP stimulation showed sensitivity 73.3% for baseline and 80% for post-stimulation results, while oCRH had sensitivity 93.3% and 100% respectively. Central-to-peripheral ACTH ratios were similar across groups. No major adverse events were reported, and both tests were well tolerated.

**Conclusion:**

Although the DDAVP stimulation test demonstrates lower diagnostic accuracy compared to the CRH test, it still provides sufficient sensitivity and given its availability and lower cost, it represents a pragmatic alternative to CRH stimulation.

**Significance statement:**

The diagnosis of pediatric CD is challenging due to the rarity of the condition and limited access to dynamic testing agents such as ovine corticotropin-releasing hormone (oCRH). This study provides the largest pediatric evaluation of the desmopressin (DDAVP) stimulation test, demonstrating its diagnostic accuracy and safety profile comparable to oCRH stimulation. The findings support the use of DDAVP as a reliable and practical alternative for diagnosing CD in children, particularly in settings where oCRH is unavailable. This work addresses a critical gap in pediatric endocrinology and has the potential to improve diagnostic pathways and outcomes in this population.

## Introduction

ACTH-secreting pituitary adenomas (PAs) causing Cushing’s disease (CD) represent the majority (~ 70–80%) of cases of endogenous Cushing’s syndrome (CS) in children older than 5 years of age [1, 2]. The diagnosis of CS can be complex and often requires multiple dynamic tests [3]. The Pituitary and Endocrine Societies recommend a stepwise diagnostic approach for suspected CS, starting with screening tests and proceeding to localization studies if hypercortisolism is confirmed [4, 5]. However, variability in assay performance, limited test availability, and the high incidence of incidental findings (such as pituitary incidentalomas), continue to pose challenges in selecting the most appropriate diagnostic tools and interpreting their results.

For patients with ACTH-dependent hypercortisolism, the diagnostic workup focuses on localizing the source of ACTH excess, most commonly a PA, though ectopic ACTH-secreting neuroendocrine tumors are also possible [5]. This becomes particularly challenging when pituitary MRI fails to reveal a visible tumor, which may occur in up to one third of patients [6]. ACTH-secreting PAs express receptors for corticotropin-releasing hormone (CRH) and administration of ovine-CRH (oCRH) stimulates ACTH and cortisol release [7]. The oCRH stimulation test, performed either in peripheral sampling or during bilateral inferior petrosal sinus sampling (BIPSS), has long been validated as a minimally invasive method to differentiate CD from ectopic sources of ACTH [8, 9]. However, the discontinuation of manufacturing of oCRH in the United States and the lower cost of alternative stimulants such as desmopressin (DDAVP, a synthetic arginine vasopressin analogue) have led to increased use of the DDAVP stimulation test.

DDAVP stimulates ACTH release via AVPR1b receptors found in corticotroph PAs, but not typically expressed in normal pituitary tissue or ectopic ACTH-secreting tumors [10]. Therefore, a rise in ACTH and cortisol following DDAVP is suggestive of CD [11, 12]. However, the lack of pediatric specific safety and accuracy data limit its use in the pediatric population.

In this study we describe the procedure of DDAVP stimulation test performed with peripheral sampling or in the context of BIPSS, and we compare its performance with the CRH test in a pediatric cohort.

## Methods

### Study design and patient selection

This was a single-center, retrospective study conducted at the *Eunice Kennedy Shriver* National Institute of Child Health and Human Development (NICHD) in Bethesda, Maryland. All patients were enrolled under an IRB-approved protocol (Protocol ID: NCT 00001595) and they were evaluated at the National Institutes of Health (NIH) Clinical Center (CC). Written informed consent was provided by all parents and assent by pediatric patients if developmentally appropriate for all research procedures.

We identified patients with a final diagnosis of CD who have undergone peripheral DDAVP stimulation test (*n* = 32) or BIPSS with DDAVP stimulation (*n* = 15) between 2021 and 2025 (DDAVP group). We then reviewed our historic cohort and identified patients who have undergone peripheral oCRH stimulation test or BIPSS with oCRH stimulation during their diagnostic workup, matched 1:1 for age, sex and tumor size (CRH group).

The diagnosis of CS was based on clinical features and standard biochemical testing, including a 1 mg (or weight-based adjusted dose) overnight oral dexamethasone suppression test, late-night serum cortisol, and/or 24-hour (24h) urinary free cortisol (UFC), in accordance with current guidelines and adjusted for the pediatric population [3, 5]. All patients were eventually diagnosed with CD either by histologic confirmation of the diagnosis on the resected tumor, or clinical and biochemical remission after transsphenoidal surgery (TSS). Demographic, clinical, biochemical, imaging, surgical, and histopathology data were collected for analysis. Tumor size was recorded based on the MRI report, or if no adenoma was reported at the MRI, tumor size was recorded as 0.5 mm since the thinnest slice of the images we obtain are 1 mm, acknowledging that this assumption could underestimate the size of a larger tumor which lacked radiographic characteristics to be distinguished in the MR images. Cortisol was measured with solid-phase, competitive chemiluminescent enzyme immunoassay (CMIA) on Siemens Immulite 2500 analyzer (Malvern, PA) until 2020 and on Abbott Architect from 2020 until 2025. ACTH was measured with CMIA on Siemens Immulite 2500 analyzer until 2012 and on Immulite 200 XPi analyzer from 2012 until 2025. UFC was measured with chemiluminescent enzyme immunoassay until 2011 and with High Performance Liquid Chromatography/Tandem Mass Spectrometry since 2011 (LC-MS/MS). UFC is reported as both absolute values (mcg/24h) and as the fold change from the upper limit of normal (ULN), to account for variable reference range per age and assay.

### Peripheral stimulation test

Patients were admitted at the inpatient pediatric floor of NIH CC at least one day prior to the procedure. An intravenous (IV) catheter was placed in the forearm at least one hour prior to the test initiation (most commonly 1–2 days prior to testing). Patients were fasting and remained lying in bed for the duration of the test. Samples were collected at times − 15 and 0 min prior to administration of stimulant at approximately 8:00am.

In the DDAVP group, 10mcg of DDAVP (2.5mL of 4mcg/mL solution) was administered via IV push over 30 s, followed by a 2mL normal saline flush. Samples were then collected at additional timepoints after administration of DDAVP at + 15, +30, + 45, and + 60 min. In a subset of patients, samples were collected at + 10, +20, + 30, +45, and + 60 min but results were not considered significantly different and eventually protocol was adjusted to sampling every 15 min. For this subset of patients (*n* = 7) the highest value of samples at + 10 and + 20 min was used as the + 15 min value. Patients were advised to follow moderate fluid restriction after DDAVP administration (max 40oz/1.2 L) for 24 h post-procedure, unless otherwise indicated by the treating physician. Intake/output monitoring was recommended for 24 h, and a repeat basic metabolic panel was obtained the following day.

In the CRH group, after baseline samples were obtained, patients received 1mcg/kg, max 100mcg, of oCRH via IV push, and samples were collected at times + 15, +30, and + 45 min after administration [3]. 

Samples were analyzed for cortisol and ACTH and the percentage change from baseline was calculated as: [(peak level after stimulation – baseline level)/baseline level]*100. The DDAVP test was considered consistent with CD based on previously published criteria: >18% increase in cortisol and >33% increase in ACTH [12]. The CRH test was considered consistent with CD if there was >20% increase in cortisol and >35% increase in ACTH [3].

### Bilateral inferior petrosal sinus sampling (BIPSS)

BIPSS was performed based on standard protocols by an interventional radiologist under anesthesia as previously described [13]. Briefly, catheters were advanced to bilateral petrosal sinuses via radiological guidance through femoral veins. Blood samples were collected at all timepoints simultaneously from each of the petrosal sinus catheter (right, left) and peripheral samples drawn from a vascular catheter introducer sheath in a femoral vein. Baseline samples were collected at − 5 and 0 min. In the DDAVP group, after collecting samples at time 0 min, 10mcg of DDAVP (2.5mL of 4mcg/mL solution) was administered as an IV push over 30 s, followed by a 2mL normal saline flush. In the CRH group, after collecting samples at time 0 min, 1mcg/kg, max 100mcg, of oCRH was administered as an IV push over 30 s via peripheral IV catheter. Post-stimulation blood samples were collected at + 3, +5, and + 10 min. Patients who received DDAVP were advised to follow moderate fluid restriction as described above. Test results were considered consistent with CD if the baseline central:peripheral (C:P) ACTH ratio was >2 and/or the stimulated C:P ratio >3 [14–16].

### Statistical analysis

Baseline characteristics were summarized using descriptive statistics. Non-normally distributed data are shown as median [Q1, Q3] and were compared between groups with the Wilcoxon rank-sum test. Normally distributed data are shown as mean (standard deviation, SD) and were compared between groups with student’s t-test. Categorical data are shown as counts and proportions and were compared between groups using χ^2^ test or Fisher’s exact test as appropriate. To assess whether hormone levels or ratios changed significantly over time and differed between the CRH and DDAVP groups, variables were log-transformed to achieve approximately normal distribution, and two-way repeated measures analysis of variance (ANOVA) was performed with time and stimulation group as fixed effects. Area under the curve (AUC) was calculated for timepoints from 0 min to 45 min for the peripheral stimulation test, and from 0 min to 10 min for BIPSS. Sensitivity was calculated using predefined criteria, and Fisher’s exact test was employed to compare sensitivity between the CRH and DDAVP groups. Missing data were considered as missing by chance and were not replaced. A p-value < 0.05 was considered statistically significant. Analyses were conducted using R/RStudio software.

## Results

### Cohort characteristics

In the peripheral stimulation test cohort, a total of 64 patients were included, consisting of 34 males (53%) and 30 females (47%). In accordance with the selection criteria, the two groups (DDAVP and CRH) were similar in age at time of testing, tumor size, and proportions of negative MRI at diagnosis. Markers of hypercortisolemia were also similar between the two groups, including late night serum cortisol, 24h UFC, and morning ACTH levels.

In the BIPSS analysis, a total of 30 patients were included, consisting of 18 males (60%) and 12 females (40%). The two groups were similar in age at time of testing, tumor size, and proportions of negative MRI at diagnosis. Markers of hypercortisolemia were also similar between the two groups. All corresponding results have been summarized in Table 1.

**Table 1:** Characteristics of patients undergoing Desmopressin (DDAVP) and oCRH stimulation test

	Peripheral Stimulation Test			BIPSS		
Variable	DDAVP (*N* = 32)	CRH (*N* = 32)	*P*-value	DDAVP (*N* = 15)	CRH (*N* = 15)	*P*-value
Sex; Male	17 (53%)	17 (53%)	1	9 (60%)	9 (60%)	1
Age (years)	13.1 (3.4)	13.2 (3.2)	0.97	12.0 (3.6)	12.0 (3.4)	1
Initial diagnosis	27 (84%)	31 (97%)	0.20	14 (93%)	14 (93%)	1
Disease duration (years)	2.5 [1.8, 3.0]	3.0 [1.6, 3.3]	0.35	2.0 [1.5, 2.5]	2.7 [1.1, 3.4]	0.46
Tumor size (mm)	4.0 [0.5, 6.1]	5.0 [0.5, 6.3]	1	0.5 [0.5, 1.8]	0.5 [0.5, 2.0]	0.98
MRI Negative	11 (34.4%)	12 (37.5%)	1	11 (73.3%)	11 (73.3%)	1
Midnight serum cortisol (mcg/dl)	13.2 [10.9, 17.0]	13.9 [12.6, 20.3]	0.13	14.1 [12.3, 17.1]	12.6 [11.1, 17.2]	0.56
UFC (mcg/24h)	170 [117, 396]	175 [132, 306]	0.95	140 [120, 345]	175 [145, 307]	0.27
UFCxULN	3.6 [2.5, 8.3]	4.2 [2.2, 6.5]	0.67	3.4 [2.7, 6.7]	4.5 [3.4, 6.4]	0.65
Morning ACTH (pg/ml)	43.5 [31.0, 51.1]	40.8 [33.4, 52.3]	0.92	33.0 [24.8, 47.1]	32.5 [20.6, 36.5]	0.45
Cortisol change during stimulation test (%)	58.2 [40.6, 85.7]	82.3 [44.1, 110.0]	0.38	NA	NA	NA
AUC for cortisol	848 [730, 1000]	1350 [1080, 1560]	< 0.001	NA	NA	NA
ACTH change during stimulation test (%)	122.0 [53.5, 206.0]	188.0 [117.0, 342.0]	0.065	NA	NA	NA
AUC for ACTH	3110 [2390, 3890]	5430 [3170, 9370]	< 0.001	NA	NA	NA
Baseline Central:Peripheral ACTH	NA	NA	NA	7.49 [4.16, 20.2]	5.59 [2.13, 10.6]	0.20
Stimulated Central:Peripheral ACTH	NA	NA	NA	18.5 [12.3, 65.4]	17.1 [8.88, 22.1]	0.37
AUC for Central:Peripheral ACTH	NA	NA	NA	104 [66.8, 151]	121 [91.5, 408]	0.21

Results presented as counts (percentages), mean (SD) or median [Q1, Q3]

ACTH: Adrenocorticotropic Hormone, AUC: Area under the curve, BIPSS: Bilateral Inferior Petrosal Sinus Sampling, UFC: Urinary Free Cortisol; UFCxULN: UFC -fold change to the upper limit of normal

### Stimulation test results

In the peripheral stimulation test results, although screening markers of hypercortisolemia were overall similar between the two groups, baseline serum cortisol at the time of the stimulation test was lower in the DDAVP group (14.1 mcg/dL [12.4, 16.8]) compared to the CRH group (18.7 mcg/dL [15.5, 24.7], *p* < 0.001), while baseline ACTH levels were similar between the two groups (47.5 pg/mL [27.5, 58.0] in the DDAVP group vs. 48.5 pg/mL [33.4, 58.6] in the CRH group, *p* = 0.73).

**Fig. 1:**
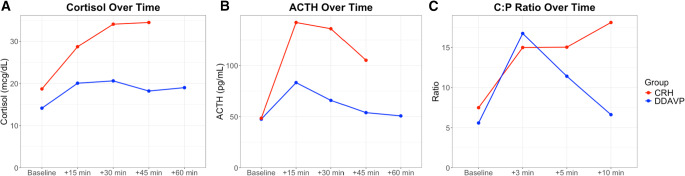
Hormonal responses to desmopressin (DDAVP) and ovine corticotropin-releasing hormone (oCRH) stimulation in pediatric Cushing’s disease. (**A**) Serum cortisol concentrations over time following peripheral stimulation, (**B**) Plasma ACTH levels over time following peripheral stimulation, (**C**) Central: Peripheral, (C:P) ACTH ratios over time during bilateral inferior petrosal sinus sampling, (BIPSS). Lines follow median values at each timepoint for CRH (red) or DDAVP (blue) group

In the repeated measures analysis of cortisol during the stimulation test, significant main effect of time (*p* < 0.001) and group (*p* < 0.001) were noted, suggesting that cortisol values were significantly different over time and between the two groups. The time-by-group interaction was also significant (*p* = 0.031), suggesting that the pattern of change over time also differed. Furthermore, AUCs were also different between the two groups, with the CRH group having a higher AUC compared to the DDAVP group (*p* < 0.001). In the analysis of ACTH between the two groups, there was significant effect of time (*p* < 0.001), but group (*p* = 0.05), and the time-by-group (*p* = 0.11) interaction did not reach statistical significance suggesting that ACTH levels changed overtime but retained a similar overall pattern between the groups. When analyzing AUCs for the ACTH secretion, the CRH group had higher AUC compared to the DDAVP group (*p* < 0.001). Peak cortisol levels occurred more frequently at 30 min in the DDAVP group and at 45 min in the CRH group, while peak ACTH levels occurred more frequently at 15 min post-stimulation in both groups (Fig. 1)

**Fig. 2:**
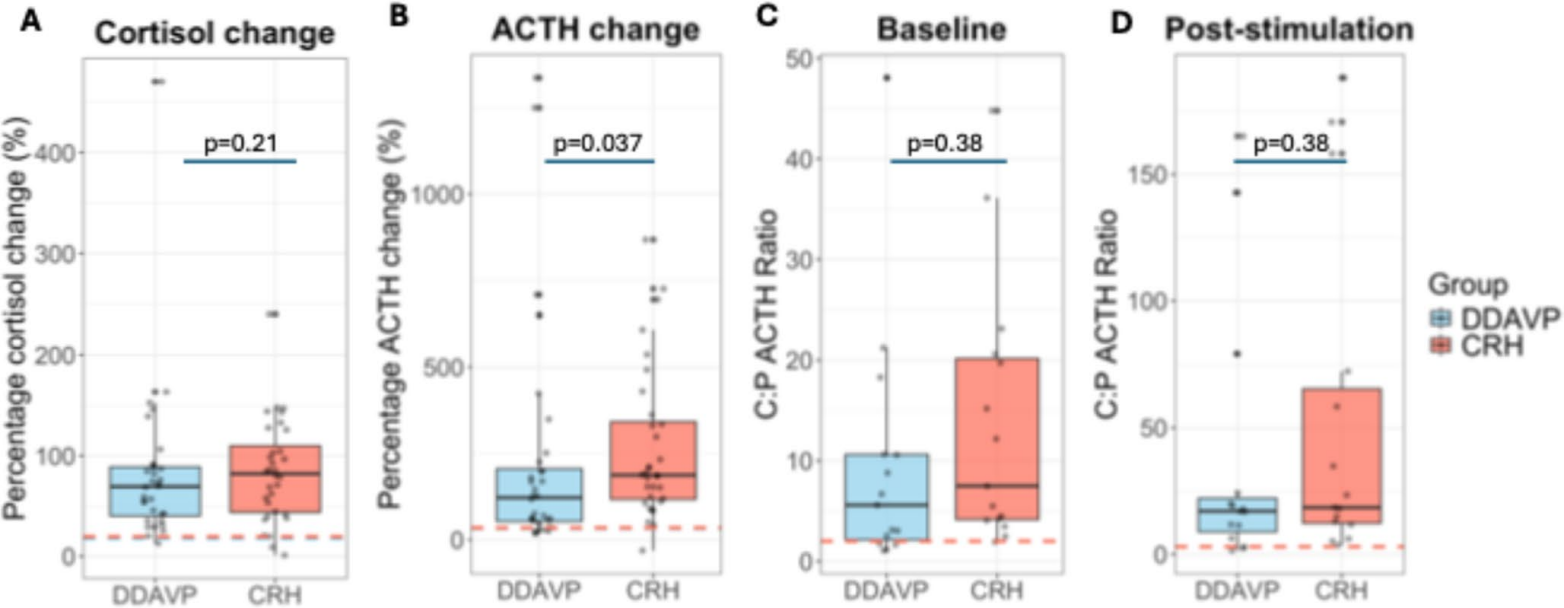
Group comparison of cortisol and ACTH levels in response to desmopressin (DDAVP) and ovine corticotropin-releasing hormone (oCRH) stimulation in pediatric Cushing’s disease. Percentage change from baseline in cortisol (**A**) and ACTH (**B**) levels following peripheral stimulation. Central:Peripheral (C:P) ACTH ratios at baseline (**C**) and post-stimulation (**D**) during bilateral inferior petrosal sinus sampling (BIPSS). Box plots display medians, interquartile ranges, and 1.5× IQR whiskers. Dashed lines represent diagnostic thresholds

When looking into the percentage change from baseline, the median percentage change in cortisol was 58.2% [40.6, 85.7] after DDAVP and 82.3% [44.1, 110.0] after oCRH stimulation (*p* = 0.21), while the median percentage change in ACTH was 122% [53.5, 206.0] and 188.0% [117.0, 342.0] after DDAVP and oCRH stimulation respectively (*p* = 0.037, Fig. 2).

In the BIPSS stimulation test, the repeated measures analysis noted that in both groups there was significant effect of time (*p* < 0.001), but group and time-by-group interaction did not differ (*p* > 0.05), suggesting that overall, there was a similar change of the C:P ACTH ratios between the two groups post-stimulation (Fig. 1). AUCs for C:P ratios did not reach statistical significance between the two groups (*p* = 0.21). Peak ratios occurred more frequently at 3 min post-stimulation in both groups. Baseline C:P ACTH ratios were similar between the two groups (5.6 [2.1, 10.6] in the DDAVP group vs. 7.5 [4.2, 20.2] in the CRH group, *p* = 0.20) and the maximum C:P ratios post-stimulation remained similar (17.1 [8.9, 22.1] in the DDAVP group vs. 18.5 [12.3, 65.4] in the CRH group, *p* = 0.37, Fig. 2).

### Sensitivity analysis

**Fig. 3:**
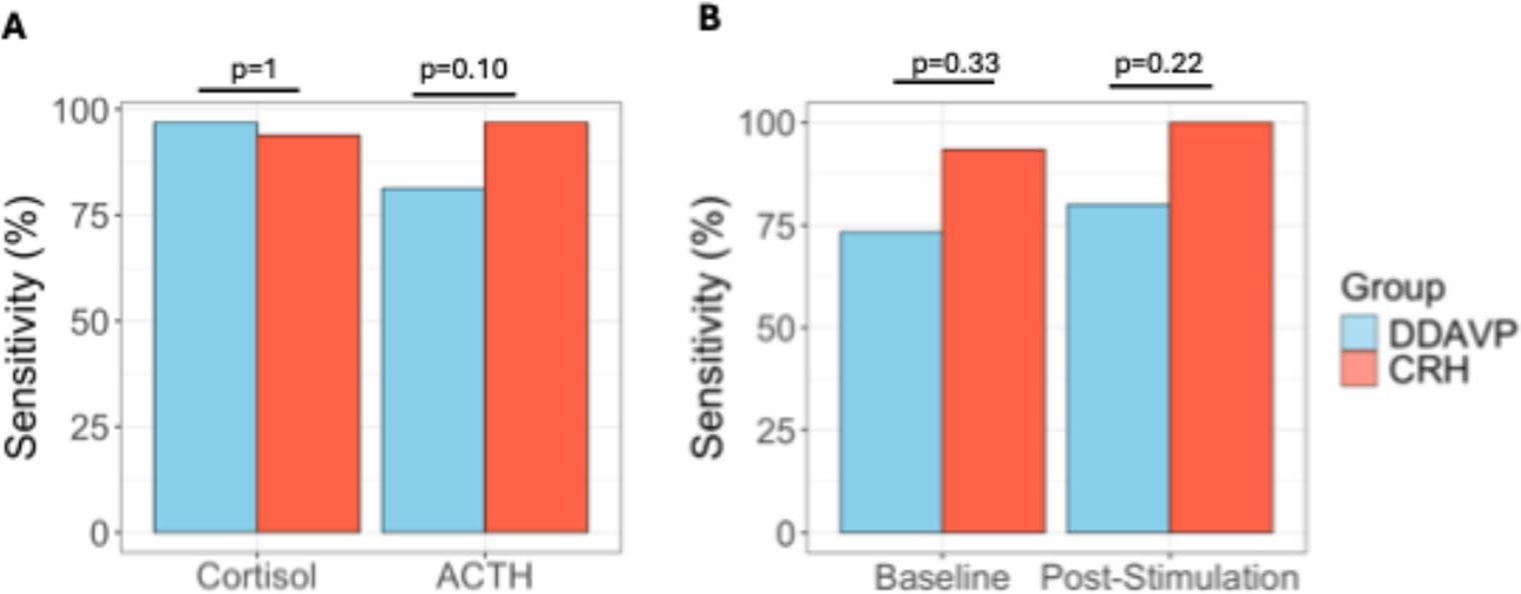
Sensitivity of desmopressin (DDAVP) and ovine corticotropin-releasing hormone (oCRH) stimulation tests in pediatric Cushing’s disease. Sensitivity for cortisol and ACTH results in the peripheral stimulation test (**A**); and the Central:Peripheral (C:P) ACTH ratio in bilateral inferior petrosal sinus sampling (**B**)

Using the diagnostic thresholds specified above for the peripheral stimulation test, sensitivity in the DDAVP group was 96.9% for cortisol and 81.3% for ACTH. In comparison, the CRH group showed 93.8% sensitivity for cortisol and higher sensitivity for ACTH (96.9%, Fig. 3), which however did not differ statistically between the two groups (*p* > 0.05).

Eight patients (12.5%) had a false negative response in either cortisol (range of percentage change of cortisol from baseline: 1–13%) or ACTH (range of percentage change of ACTH from baseline: −31-30%). Of these, six patients underwent DDAVP and two patients had oCRH stimulation test. Only two patients had inadequate response in both cortisol and ACTH and would have been misclassified (one in each of the DDAVP and CRH groups). Of the seven patients with false negative stimulation tests, only one was documented to have a hemolyzed blood sample. All patients who underwent DDAVP stimulation had an ACTH response of > 19%.

In the BIPSS analysis, using the thresholds above, sensitivity was 73.3% and 93.3% in the DDAVP and CRH group respectively (*p* = 0.33) for the baseline ratios, and 80% and 100% in for the stimulated ratios (*p* = 0.22, Fig. 3). Five (5) patients showed an inadequate response at baseline and three of them also had a false low response post-stimulation. All three of the patients who failed both the baseline and the post-stimulation cutoffs, and who would have been misclassified as possible ectopic CS, were in the DDAVP group and were previously described in a study of negative BIPSS results [13]. Two of these three patients were noted on venograms to have poorly developed inferior petrosal veins that suggested BIPSS might not yield reliable results.

### Side effects

Overall patients tolerated the procedures without significant adverse events or complications. One patient in the CRH group reported mild headache, which resolved spontaneously within a few hours. No episodes of hyponatremia or venous thrombosis were recorded.

## Discussion

The diagnostic workup of CS can be challenging and complex involving many baseline and dynamic tests. Especially in the pediatric population, the rarity of the disease makes the diagnostic process more difficult. We herein present the DDAVP stimulation test in pediatric patients with CD. We report the performance of the test which although lower than oCRH, still yields sufficient sensitivity overall without significant side effects.

DDAVP stimulation test is used for the differential diagnosis of pituitary versus ectopic sources of hypercortisolemia in ACTH-dependent CS. Although rare in children, infrequent cases of ectopic pediatric CS have been reported, some of which have led to devastating results, even death [2, 17, 18]. In a recent study on a non-invasive approach to differential diagnosis of ACTH-dependent CS, Frete et al. incorporated the CRH or DDAVP stimulation test in their diagnostic algorithm [12]. We have also recently described that in our cohort, pediatric patients with positive high dose dexamethasone suppression test and peripheral CRH or DDAVP stimulation test consistent with pituitary source ended up having CD irrespective of the MRI findings [2]. This could suggest that in this population, especially when IPSS would delay evaluation or is not available, DDAVP stimulation test can assist in important decisions for the management of the patient. The sensitivity of DDAVP test was lower than that of CRH for the diagnosis of CD. Similar findings were noted in a metanalysis by Ceccato et al., where CRH showed higher sensitivity than DDAVP [19]. In the absence of oCRH in some countries, DDAVP remains a safe and effective alternative for this patient population.

The utility of DDAVP stimulation test expands beyond the differential diagnosis of ACTH-dependent CS. Studies have shown that it can be used for the differential diagnosis of non-malignant hypercortisolism as well as a marker of post-operative remission, which have not been explored in this study [20, 21].

False negative and recently false positive results to DDAVP stimulation test have been reported in the literature. Although initially thought that AVPR1b receptors are located only on corticotroph tumors, some ectopic tumors show response [22]. Our false negative results are contingent to the cutoff values we used in this study. For the peripheral stimulation test, we used the latest cutoff values suggested by Frete et al. Initial descriptions of the test suggested the use of >50% change for ACTH and >20% change for cortisol, which would lead to 8 patients in our group being misclassified (instead of 6 with the current criteria). However, if we use a combination of either cortisol or ACTH response, then one patient would still be misclassified due to inadequate response to both cortisol (13%) and ACTH (26.6%). A threshold for ACTH of >20% would have identified all patients with CD in our cohort, but would potentially yield false positive results in patients with ectopic CS. In the BIPSS interpretation, we used the cutoff criteria of >2 for baseline and >3 for stimulated values. Recently a study suggested that cutoffs of C:P ACTH ratio >1.4 for baseline and >2.8 for post-stimulation results would yield better accuracy [23]. If we used those cutoffs, then one patient at baseline and two patients at post-stimulation would have been misclassified. Only one patient would have both ratios below the cutoff criteria (baseline: 1.1, post-stimulation: 1.5) and eventually be considered as ectopic CS. In the pediatric population, one major factor to consider is that technical limitations may pose difficulties in reaching the petrosal sinuses; in these cases, results are not dependent on the stimulant used.

Additional possible explanations for negative results in patients with CD may be a cyclical pattern of cortisol secretion. We have ruled out this possibility in our patients since all of them had midnight serum cortisol the night(s) prior to the test and all were elevated suggesting they had active hypercortisolemia. Technical difficulties could explain some cases where administration of the medication may not have been complete or appropriately delivered. However, most patients had decreased urine output as evidence of an effective dose of DDAVP in their circulation. Finally, as tumors may have pulsatility on ACTH secretion, it is possible that the test coincided with an endogenous pulse of the tumor ACTH secretion that further masked the effect of DDAVP administration.

Certain limitations exist for this study. We had limited patients with ectopic CS and thus we could not compare the performance of the test between patients with CD and patients with ECS. However, we did not aim to define the diagnostic cutoffs of each test but rather to assess the safety and concordance to the CRH stimulation test. Furthermore, ectopic CS is quite rare in the pediatric population that it would be very difficult to recruit enough patients. Although this is the largest to our knowledge cohort of pediatric patients with DDAVP stimulation test, the number of patients may still have been too low to detect significant differences between the tests. Finally, the historic CRH group were evaluated with variable cortisol and ACTH assays which may affect the comparison of absolute values between the two groups. Thus, we present our results also as percentage changes to compare the performance of the tests.

In conclusion, we show the performance of DDAVP stimulation test performed either peripherally or during BIPSS. The test is well tolerated, and no significant side effects were noted. The test shows comparable sensitivity and a valid alternative to the oCRH stimulation test, in the absence of this agent.

## Data Availability

All raw data used in this study will be deposited in data repository listed in References [24].

## Declarations

### ClinicalTrials.gov ID

NCT00001595.
